# The path towards polio eradication over 40 years of the Expanded Program on Immunization in the Americas

**DOI:** 10.26633/RPSP.2017.154

**Published:** 2017-12-20

**Authors:** Cristina Pedreira, Elizabeth Thrush, Gloria Rey-Benito, Ana Elena Chévez, Barbara Jauregui

**Affiliations:** 1 Immunization Unit, Pan American Health Organization Regional Office of the World Health Organization Washington, DC United States of America Immunization Unit, Pan American Health Organization, Regional Office of the World Health Organization, Washington, DC, United States of America.

**Keywords:** Poliomyelitis, immunization programs, mass vaccination, global health, Americas., Poliomielitis, programas de inmunización, vacunación masiva, salud global, Américas., Poliomielite, programas de imunização, vacinação em massa, saúde global, Américas.

## Abstract

*This article synthesizes the important lessons learned from polio eradication in the Region of the Americas, including initial and more recent challenges and best practices, as well as particular factors surrounding attainment of this ambitious goal. Using documents, interviews, and country surveys, the authors describe and analyze the strategies and lessons learned during the 40 years of the Expanded Program on Immunization (1977 – 2017). Some major milestones and chxallenges specifically covered are: the Vaccine-derived Poliovirus (VDPV) outbreak in the Dominican Republic; the regional “mop-up operation;” poliovirus containment in essential facilities; the unprecedented introduction of inactivated polio vaccine (IPV); the synchronized switch from trivalent to bivalent OPV; and the countries’ unfailing commitment to the cause*.

## RESUMEN

En este artículo se sintetizan las importantes enseñanzas extraídas de la erradicación de la poliomielitis en la Región de las Américas. Se presta especial atención a los retos y las mejores prácticas que se abordaron en un principioy a los más recientes, así como a los factores particulares que han girado en torno al logro de esta meta de gran alcance. Por medio de documentos, entrevistas y encuestas nacionales, las autoras describen y analizan las estrategias y enseñanzas extraídas a lo largo de los 40 años del Programa Ampliado de Inmunización (1977–2017). Entre los hitos y retos más importantes que se abordan en el artículo se encuentran los siguientes: el brote del poliovirus derivado de la vacuna en la República Dominicana; la campaña de vacunación “de seguimiento” a nivel regional; la contención del poliovirus en las instalaciones esenciales; la introducción sin precedentes de la vacuna antipoliomielítica con virus inactivados (VPI); el cambio sincronizado de la vacuna antipoliomielítica oral trivalente por la vacuna antipoliomielítica oral bivalente (conocido como “el switch”); y el compromiso inquebrantable de los países con esta causa.

## RESUMO

Este artigo sintetiza os principais ensinamentos da erradicação da poliomielite na Região das Américas, expondo as dificuldades enfrentadas ao início e em um período mais recente, as boas práticas e os fatores inerentes relacionados ao alcance desta meta ambiciosa. A partir de documentos, entrevistas e levantamentos nos países, os autores descrevem e analisam as estratégias empregadas e a experiência obtida ao longo dos 40 anos do Programa Ampliado de Imunização (1977–2017). Entre os marcos e os desafios importantes abordados estão: o surto por vírus da poliomielite derivado da vacina (VDPV) na República Dominicana, a operação regional de “acabamento”, a contenção do poliovírus em laboratório, a introdução inédita da vacina contra poliomielite com vírus inativado (VPI), a transição sincronizada da vacina oral contra poliomielite trivalente à bivalente e o firme compromisso dos países com a causa.

The elimination of smallpox from the Region of the Americas in 1971 was an unprecedented effort led by the Pan American Health Organization (PAHO) as part of a worldwide drive coordinated by the World Health Organization (WHO). Three years later, in 1974, the Expanded Program on Immunization (EPI) was created by the World Health Assembly (1). EPI was then endorsed in the Americas in 1977 by the PAHO Directing Council (2), which concurrently launched the PAHO Revolving Fund for Vaccine Procurement to provide Member States with an effective mechanism for procuring vaccines at the lowest market price (2). In the same year, the world saw its last case of smallpox, and the disease was declared eradicated in 1979. The success against smallpox signaled the feasibility of disease eradication and provided a model to achieve it (3). Together with the creation of the EPI and the PAHO Revolving Fund, it set the foundation for the successful eradication of polio in the Americas.

A literature search was conducted of PubMed Central® (U.S. National Library of Medicine, Bethesda, Maryland, United States) and IRIS (PAHO/WHO Institutional Repository, Washington, DC, United States) for documents on polio eradication in the Americas published in 1976 – 2017. The results included PAHO and WHO resolutions and progress reports; published papers on polio eradication in the Americas; and reports and evaluations from independent expert groups, such as the International Commission for the Certification of Poliomyelitis Eradication (ICCPE), which declared the Region as poliofree, and the Taylor Commission, which evaluated the impact of polio eradication on health systems.

## EARLY SIGNS THAT ERADICATION WAS POSSIBLE

In 1969 – 1984, there were 53 251 cases of poliomyelitis reported by the 46 countries of the Americas— likely underestimated due to inadequate reporting (4). The number of reported cases of paralytic polio decreased from 4 728 in 1979 to 525 in 1984 (a 90% reduction); 26 countries of the Region had achieved control of the disease (4). This remarkable decline was due to acceleration in immunization activities and increases in vaccination coverage (4).

Cuba was the first country to show that eradication was possible. In 1959 – 1961, Cuba reported 1 162 cases of poliomyelitis (5). In 1962, Cuba launched a nationwide annual polio vaccination campaign with oral polio vaccine (OPV) as part of a comprehensive national polio elimination program. The campaign targeted children 1 month - 14 years of age, with two doses given 4 weeks apart; it was implemented only twice a year (3). This innovative campaign and fol-low-ups were characterized by high vaccination coverage achieved through week-long drives, surveillance of suspected cases, and outbreak investigation and control, when necessary. This strategy effectively halted polio transmission in Cuba, with the last case being recorded in May 1962 (6).

**Figure 1.**
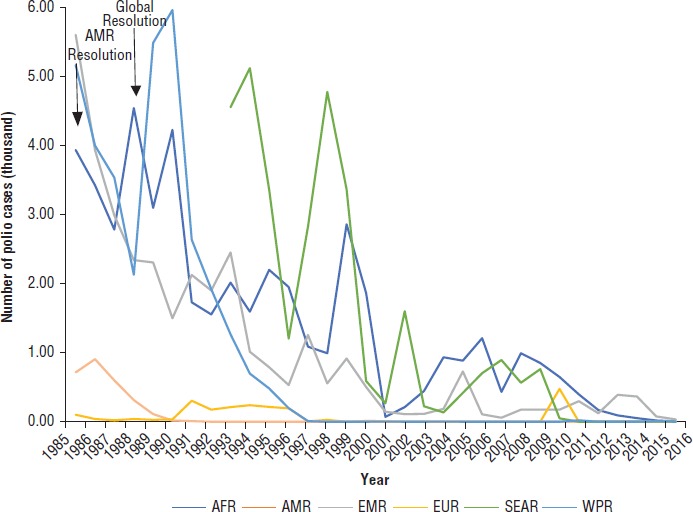
Number of confirmed cases of poliomyelitis, by Region of the World Health Organization,^a^ and timing of the resolutions for polio eradication, 1985^b^ – 2016

^a^ AFR = African Region; AMR = American Region; EMR = Eastern-Mediterranean Region; EUR = European Region; SEAR = South-East Asian Region; WPR = Western Pacific Region.

^b^ Numbers of polio cases in SEAR are since 1993; from 1980 – 1994, numbers ranged from 6 470 – 39 936 annually.

***Source:*** World Health Organization. Available from: http://www.who.int/immunization/monitoring_surveillance/data/en Accessed 14 September 2017.

Brazil became the second country to pave the way for polio eradication in the Region. During the 1970s, Brazil was reporting 2 000 – 3 000 cases of polio annually, with known under-reporting due to a weak dis-ease surveillance system (4). In 1980, recognizing that polio was a major public health problem and considering the experience in Cuba, Brazil developed a strategy of two National Polio Immunization Days per year to increase coverage (4). Key components of this strategy included high vaccination coverage (approached 100%), active case follow-ups, and the creation of a polio laboratory network. Political commit-ment, the availability of national resources, the engagement of partners and recruitment of nearly 300 000 volunteers made it possible to vaccinate 20 million children in a single day. After the implementation of these polio vaccination days, the reported incidence of the disease dropped dramatically, from a rate of 2.2 per 100 000 in 1979 to 0.062 in 1984 (4).

Other countries took note and began enhancing their polio immunization programs, particularly, Bolivia, Colombia, Dominican Republic, El Salvador, Mexico, and Nicaragua (7). In 1980, Colombia implemented an innovative strategy named “canalization” by which community leaders sought out unvaccinated children and brought them to either mobile or fixed vaccination posts (4). This strategy was important because it allowed for a rapid increase in vaccination coverage. In 1984, Colombia conducted National Immunization Days in June, July, and August, with strong support from international organizations and the involvement of all sectors of society. Mexico, in turn, implemented a strategy that mobilized the community through local health committees; these promoted vaccination to help improve coverage (4).

These countries’ strategies and successes, and in particular, the experience of Brazil, were noticed by the head of the Immunization Unit at PAHO, Ciro de Quadros, and by Joao Baptista Risi, a Brazilian veteran of the smallpox eradication program. To them, these accomplishments indicated that a well-planned, disease-specific, Regional program could eliminate polio from the Americas. They also saw that a goal as inspiring as this one could garner additional political and public support for the EPI and surveillance efforts overall (3, 8). In late 1984, de Quadros received a green light from PAHO leaders to begin a coordinated Regional effort to eliminate polio from the Americas (3).

## THE COMMITMENT OF THE AMERICAS

In September 1985, PAHO Member States unanimously adopted a Directing Council Resolution to eradicate the indigenous transmission of wild poliovirus in the Americas by 1990 (9). A Regional polio vaccination strategy was adopted and national immunization days (NIDs), modeled after the previous successes, were added to routine vaccination programs (10). The PAHO Director also established a Technical Advisory Group (TAG)—composed of regional experts in disease control—to review the Plan of Action for the eradication of indigenous transmission of wild poliovirus in the Americas (11, 12). This was a leap forward for EPI in the Americas, given that TAG is now a leading expert group that helps set priorities and makes recommendations on all vaccine-preventable diseases for the Region. Additionally, an interagency coordinating committee was formed with participants from the Inter-American Development Bank, the United Nations International Children’s Emergency Fund (UNICEF), the United States Agency for International Development (USAID), Rotary International, and the Task Force for Child Survival, all of which endorsed and strongly supported the Plan of Action (11).

The objectives of the Plan of Action were to promote the overall development of the EPI; to speed up attainment of its objectives; to set up regional and national surveillance systems; and to eradicate indigenous transmission of wild poliovirus from the Region by 1990. Through the Regional surveillance system, all cases of acute flaccid paralysis (AFP) were immediately investigated to discard or confirm a polio diagnosis, and measures to stop transmission were rapidly implemented (13). This prompted special efforts to detect wild poliovirus, as well as house-to-house “mop-up” campaigns that vaccinated all children in the neighborhood immediately surrounding any case of wild poliovirus infection.

In its first year, the Plan of Action was met with overwhelming support from all countries and collaborating agencies, which generated the momentum needed for success (14). All countries except one reported weekly AFP cases to PAHO; and PAHO, in turn, provided assistance in case investigation and control measures.

### Scaling up of vaccination activities

The main strategy for interrupting wild poliovirus transmission was the implementation of NIDs twice a year, targeting children less than 5 years of age with one dose of trivalent oral poliovirus vaccine (tOPV) (15). This strategy was recommended as an ad hoc measure, to be gradually replaced by regular immunization services performed routinely by health services.

The Regional campaign against polio was spearheaded by governments, international organizations, and nonprofits, but just as in Brazil, its success was due to the widespread support of local communities, especially the volunteers. For instance, guerillas in El Salvador and Peru not only observed cease-fires during national immunization days, they often served as vaccinators themselves—a testament to the popularity of polio vaccination efforts (3). The high coverage levels managed to interrupt wild poliovirus transmission in the Region, and became a catalyst for strengthening routine immunization programs in all the countries.

### Surveillance strengthening

Countries’ disease surveillance systems often suffer from under-reporting of cases, which is one of the main problems that needs to be addressed in an eradication effort. This under-reporting can be due to a lack of awareness on the part of health centers and professionals, insufficient access to health care, and/or inaccurate diagnoses. Whatever the cause, inaccurate surveillance can hinder the timely implementation of early control measures. Therefore, enhanced surveillance was a critical component for the success of this initiative (13).

At the beginning of the Regional effort to eradicate polio, there was an increase in the reported number of polio cases, due to both the improved surveillance system and a few polio outbreaks, including a large one in Brazil (16). A reporting system with standard procedures and definitions was established for all AFP illnesses. Very sensitive case definitions were implemented to ensure that no AFP cases were missed. Moreover, every case of AFP was regarded as a public health emergency and investigated immediately; its chain of transmission was used to determine the extent of virus circulation in the community.

### Increased laboratory support

With the decrease in the number of cases and the increase in vaccination coverage, it became more important to determine if a poliovirus isolate was a wild virus. Several countries had low laboratory confirmation rates due to gaps in the logistics required for collecting specimens and transporting them from the field to the laboratory. PAHO supported the development of logistics systems at the national level. Additionally, laboratory support networks were created to analyze stool samples and paired sera from all detected AFP cases; plus, reference laboratories were established to provide more sophisticated tests, including genetic characterization of poliovirus isolates. PAHO immediately notified all countries of any outbreaks in the Region so that traveler’s advisories could be issued (13).

### Cooperation efforts

Inter-agency, interinstitutional, and intersectoral cooperation that was used by prior health programs, was reorganized, repurposed, and applied on a broader scale. An Inter-agency Coordinating Committee (ICC) was established at the Regional level, to help organize the specific commitments of various members. Many countries followed suit, establishing national ICCs to organize efforts at the national level. This helped mobilize necessary support and the commitment of all actors involved (17). ICCs played an important role in mobilizing resources to complement existing resources at the Ministry of Health. In addition to outside financial and technical resources, the community, community groups, and private voluntary organizations, (e.g., Rotary International, religious groups, and mass media organizations) were leveraged to collaborate toward the goal. They assisted in promotional activities, distribution of supplies, and provided personnel for vaccination activities (13). The lessons learned from this experience with interagency and intersectoral cooperation have been applied since then to other health programs (17).

### Personnel training

The Plan of Action emphasized personnel training as a critical component of the program’s success. PAHO prepared training manuals and materials, and assisted countries with customizing these to fit the local context and circumstances. Training activities also bolstered the commitment of health workers and national governments to the Regional goal of polio eradication.

BOX 1. **International Commission for the Certification of Poliomyelitis Eradication (ICCPE): criteria for certification of polio eradication in the Americas, 1993**

1. Absence of virologically-confirmed indigenous poliomyelitis cases for a period of at least 3 years under circumstances of adequate surveillance
2. Verification of the absence of detectable wild polioviruses from communities as determined by testing of stools from normal children, and where appropriate, testing of wastewater from high-risk populations
3. On-site evaluation by national certification commissions appointed jointly by the Pan American Health Organization and respective Member States, composed of knowledgeable local persons and outside experts. One or two responsible ICCPE members will serve in advisory roles during this process. After the national commission considers that the criteria have been met, the information will be submitted to the full ICCPE for final certification.
4. Establishment of appropriate measures to deal with the potential importation of cases from areas that are not free of polio.

***Source:*** Pan American Health Organization. Strategies for the certification of the eradication of wild poliovirus transmission in the Americas. Bull Pan Am Health Organ. 1993;27 (3):287-96. Reprinted with permission.

BOX 2. **International Commission for the Certification of Poliomyelitis Eradication (ICCPE): surveillance quality, 1993**

1. At least 80% of all health units included in the reporting network reporting weekly
2. At least 1 acute flaccid paralysis (AFP) case per 100 000 children less than 15 years of age
3. At least 80% of cases investigated within 48 hours
4. At least 80% of all AFP cases should have two stool specimens taken for virus culture within 2 weeks of paralysis onset
5. At least 80% of all AFP cases should have stool investigations of at least five contacts.

***Source:*** Pan American Health Organization. Strategies for the certification of the eradication of wild poliovirus transmission in the Americas. Bull Pan Am Health Organ. 1993;27 (3):287-96. Reprinted with permission.

## CERTIFICATION OF POLIO ERADICATION IN THE AMERICAS

The last case of paralysis caused by wild poliovirus in the Region was detected in Peru in 1991. Early in 1994, each country formed an independent National Certification Committee (NCC) to evaluate whether or not poliovirus transmission had been interrupted in their respective countries (15, 18). The reports presented by these committees showed that the fight against polio had been successful in interrupting transmission (6, 18). In August 1994, the International Commission for the Certification of the Eradication of Polio (ICCEP) reviewed the progress reports of the NCCs and concluded that indigenous circulation of the wild virus had been interrupted, making the Region of the Americas the first to achieve this goal. The criteria required from each country by the ICCPE to verify elimination are outlined in Box 1, and the surveillance quality indicators are listed in Box 2.

### Vaccine-derived poliovirus on Hispaniola

Maintaining the elimination in the Americas required the same methods used to eliminate polio: sustaining high vaccination coverage, continuing high quality surveillance with a high quality laboratory network, and maintaining a rapid response infrastructure to handle detection of a polio case. The importance of sustaining these efforts was made evident in October 2000 when the Dominican Republic and Haiti reported two cases of AFP (19). In the Dominican Republic, the case was found in a 9-month-old female in a rural village; while in Haiti, it was in a 2-year-old girl from a town in the Northwest Department. The cases were detected through the national surveillance systems. Stool samples were sent to the PAHO Poliovirus Detection Laboratory at the Caribbean Epidemiology Center in Trinidad and Tobago. Genetic sequencing revealed that the virus associated with this outbreak was atypical, since it was derived from the oral polio vaccine, but it diverged genetically by 3% from the parent vaccine strain type 1. It was estimated that the strain had been circulating for approximately 2 years. It had recov-ered the neurovirulence and transmissibility of wild po-liovirus, which had not been in circulation since 1985 in the Dominican Republic and since 1989 in Haiti.

In response to the outbreak, the Ministries of Health of both countries, with support from PAHO and the CDC, initiated an active search to discover the extent of virus circulation, control it, and interrupt transmission. In July 2000 – July 2001, in the Dominican Republic, a total of 123 AFP cases were found, 13 of which were confirmed as poliovirus type 1; and in Haiti, 33 AFP cases, of which 8 were confirmed as circulating type 1 vaccine-derived poliovirus (VDPV) (20). Of the 21 total patients (13 from Dominican Republic and 8 from Haiti), only 1 had a record of three doses of OPV; the others were either unvaccinated (11 patients), incompletely vaccinated (7 patients), or had unknown vaccination status (2 patients). The affected areas had poor environmental sanitation and low OPV coverage; the municipality with the most cases, had coverage of 20% – 30 % among children less than 5 years of age. Gaps in coverage were accompanied by gaps in surveillance, especially in Haiti (20).

This outbreak was successfully interrupted by achieving high OPV vaccination coverage through vaccination campaigns using various strategies, such as door-to-door vaccination, canalization, and spontaneous demand. Widespread public information and social mobilization campaigns were important factors that contributed to achieving high coverage. The importance of EPI teams working with professionals to develop a quality communications campaign and a well-informed media was one of the lessons learned from this outbreak. These were key factors in getting the correct message to the public regarding the risks and the need to vaccinate, and for maintaining the public informed throughout the outbreak. Another lesson learned was the importance of taking the time to adequately prepare the intervention and the response teams, and to coordinate the support of all actors involved. This experience emphasized the need to maintain high vaccination coverage and quality surveillance in disease-free areas until global eradication is achieved (19).

## THE POLIO ENDGAME PLAN

In 1988, the 41st World Health Assembly (WHA) resolved to eradicate polio from the world by the year 2000. It also created the Global Polio Eradication Initiative (GPEI), spearheaded by WHO, Rotary International, the Centers for Disease Control and Prevention (CDC; Atlanta, Georgia, United States), and UNICEF, and now supported by key partners like the Bill and Melinda Gates Foundation (Seattle, Washington, United States) and the Gavi the Vaccine Alliance (Geneva, Switzerland; 21). After the Region of the Americas was certified as polio-free in 1994, other WHO Regions followed: the Western Pacific Region in 2000; the European Region in 2002; and the South-East Asia Region in 2014. The number of countries with endemic polio has dropped from 125 in 1988 to only 3 in 2016—Afghanistan, Nigeria, and Pakistan, with 37 cases in total (3).

Despite the global efforts, by 2011 all Regions of the world except for the Americas had suffered the reintroduction of wild poliovirus. The Independent Monitoring Board of the GPEI stated in October 2011 that the world was not on track to interrupt poliovirus transmission and, if unsuccessful, this would be the most expensive public health failure in history (22).

In response, in May 2012 the 65th World Health Assembly adopted a landmark resolution declaring the completion of poliovirus eradication a “programmatic emergency for global public health” and requested that WHO develop a comprehensive strategic plan for polio eradication (23). By January 2013, the WHO Executive Committee had approved the Polio Eradication and Endgame Strategic Plan 2013 – 2018 (24). Its goal is the complete eradication and containment of all wild, vaccine-derived, and Sabin polioviruses, while leveraging the backbone of the polio effort to deliver other health services to the world’s most vulnerable children. This Endgame Plan includes the withdrawal of all oral polio vaccines, starting with the type 2 component of the trivalent OPV in a global, coordinated switch from trivalent to bivalent OPV. The Endgame Plan also included the introduction of at least one dose of IPV into all routine immunization programs before the switch, to maintain immunity to poliovirus type 2 among the new birth cohorts (24).

### Endgame Plan in the Americas

Prior to 2013, only OPV was part of the national immunization schedules in 32 countries in the Americas. In early 2015 – 2016, all of these countries introduced at least one dose of IPV into their routine schedules before the switch. On 17 April – 1 May 2016, when the global switch took place, 36 countries in the Americas participated, simultaneously switching from tOPV to bOPV. The remarkable success of the IPV introduction and the switch in the Americas would not have been possible without the critical support of many international and regional partners, including the CDC, the Task Force for Global Health (Decatur, Georgia, United States), UNICEF, WHO Headquarters, and Gavi-the Vaccine Alliance. These agencies provided valuable support to the Region including technical and/or financial support for the decisionmaking, planning, preparation, implementation, and validation of IPV introduction and switch (25).

Regarding polio containment, in December 2014, WHO developed the third edition of a Global Action Plan (GAP III) to minimize poliovirus facility-associated risk after polio eradication and sequential cessation of OPV use, aimed at containing all polioviruses type 2: WPV2, VDPV2 and Sabin2. On the global level, this containment plan was sequential and began with the containment of cVDPV2 and WPV-type 2 by December 2015, followed by containment of Sabin poliovirus type 2 by July 2016, just 3 months after the switch to bOPV (26). However, considering that in the Americas the last WPV was detected in 1991, and that most of this Region’s countries already had destroyed or contained all WPV and VDPV, the Regional Plan shared in 2015 (Regional GAP III) promoted destruction of all wild polioviruses and VDPV, and Sabin type 2 (27). The rest of the world will finalize the containment of all wild polioviruses 1 and 3 after the certification of wild poliovirus eradication. All Sabin polioviruses type 1 and 3 will be contained after the global interruption of the use of bOPV (26).

### Challenges ahead for the Endgame Plan

One of the greatest challenges of implementing the Endgame Plan has been the limited supply of IPV. This shortage threatens the uninterrupted administration of the vaccine to the target population. To date, manufacturers have faced difficulties in scaling up production to meet the global demand. This means that countries have had to delay their vaccine introductions, have faced stock outs, or have had to change to a fractional dose schedule.

Another challenge has been the proper transfer, destruction, and/or containment of all infectious and potentially-infectious materials in poliovirus essential facilities and the certification of these facilities by national authorities. Substantial commitment, effort, and organization are required on behalf of the countries and regions to ensure proper handling of infectious materials.

BOX 3. **Essential components for a successful vaccine-preventable disease eradication and highlights from polio eradication in the Americas**

**Global and Regional levels:**

- International support of partners to ensure necessary resources and technical support
- Involvement of inter-agency coordinating committees (ICCs)
- Regional eradication efforts require a high level of coordination among the countries and the international agencies
- The international agencies should play an important role in urging the national governments to join the enterprise
- Technical cooperation should be intensified in countries with special challenges, including sending an international team for in-country placement
- Certify sustained containment of polioviruses in facilities

**National level:**

- Political will, local alliances, and community involvement are absolutely necessary to take on the ambitious goal of eradicating a disease
- The EPI staff should be strengthened through additional human resources and training
- High coverage, active surveillance, and lab networks are essential components to disease eradication
- Conducting vaccination campaigns all at once in a short period of time is beneficial both to keep the momentum going, for logistical reasons and for immunity reasons
- Conducting quick, indiscriminate campaigns (vaccinating everyone despite prior vaccination status), reaching 90% coverage, accelerates the eradication process
- Disease-specific national responsibilities must be assigned in each country to ensure appropriate coordination among vaccination, surveillance, and outbreak control activities
- Guarantee the implementation of the containment of poliovirus plan
- Involvement (or formation) of national interagency-coordination committees

***Source:*** Prepared by the authors from the study data.

Finally, the greatest challenge and simultaneously the greatest potential looking ahead, is to garner the lessons learned, the capacity built, and the infrastructure and strategies in place from the polio eradication efforts, to strengthen the immunization programs and health systems as a whole. The Taylor Commission concluded that although the polio program in the Americas had somewhat disrupted other health activities by diverting resources, it had also substantially enhanced the health services’ reputation and ties to local communities, broadened support for vaccination at every socioeconomic level, and provided a model for social mobilization and interagency cooperation for future health challenges (3, 17). Box 3 outlines lessons learned and how these learnings may be applied to other areas.

## CONCLUSIONS

Several factors made it possible for the Americas to progress toward the goal of polio eradication. These included the existence of a plan with well-defined strategies; a very high level of political commitment among governments; the dedication, commitment and exceptional work of thousands of health care workers; a high degree of community participation; the strong collaboration of various agencies and organizations; and the availability of well-managed resources under strong PAHO leadership (15). The perseverance of the EPI and the dream of eradicating a disease will hopefully continue inspiring health care workers, national authorities, and international entities until the day that global polio eradication is declared and beyond.

Lessons stemming from the experience of polio eradication in the Americas have helped shape the strategy to eliminate measles from the Region, and should be considered for future vaccine-preventable disease eradication efforts.

### Acknowledgements.

The authors would like to thank the Member States of Latin America and the Caribbean that shared their experiences through surveys and interviews, and all the international and regional partners involved in these unprecedented polio eradication and Endgame efforts to date.

### Disclaimer.

Authors hold sole responsibility for the views expressed in the manuscript, which may not necessarily reflect the opinion or policy of the *RPSP/PAJPH* and/or PAHO.
